# Intuitive and deliberate decisions can be accounted for by the same cognitive process model

**DOI:** 10.3758/s13423-025-02853-9

**Published:** 2026-02-18

**Authors:** Sarah Forst, Andreas Glöckner

**Affiliations:** 1 School of Social Sciences, University of Mannheim, L 13, 17, 68161 Mannheim, Germany; 2 Department of Psychology, University of Cologne, Cologne, Germany; 3 Max Planck Institute for Research on Collective Goods, Bonn, Germany

**Keywords:** Decision-making, Parallel Constraint Satisfaction, Intuition, Dual-process models, Computational modeling, Heuristics

## Abstract

A central open question in research on intuitive and deliberate cognitive processing is whether both can be captured by adaptations within a single cognitive mechanism or require distinct computational processes. We test whether interactive activation processes, proposed as general models of cognition, can account for both intuitive and deliberate decisions. In an online experiment (*N* = 128), we analyzed the effects of decision-mode instructions in a probabilistic inference task using a computational modeling approach. The manipulation of decision mode was successful as indicated by substantial changes in decision time and subjective experience of conscious decision-making. The manipulation, however, did not influence the distribution of decision strategies. There was no indication that more serial, rule-based, as opposed to holistic, associative (i.e., coherence-based) processes were used under a deliberation instruction. In both conditions, a Parallel Constraint Satisfaction (PCS) model for decision-making, which is based on interactive activation processes, accounted best for the data for the majority of participants. Deliberation increased the quality of the choices measured as adherence to a rational standard. In the deliberation mode, the observed patterns of response times and confidence were more in line with the predictions of the PCS model than under an intuitive instruction. Our results are consistent with an integrated processes perspective, suggesting that coherence-based mechanisms can account for behavior under both intuitive and deliberate decision modes.

In human thinking, some cognitive operations unfold quickly and effortlessly, while others require slow, effortful reflection. Popular dual-process theories explain these patterns by distinguishing between intuitive (or System 1) and deliberate (or System 2) processes or modes of thought (e.g., Evans, 2008; Evans & Stanovich, 2013; Sloman, 1996; Kahneman & Frederick, 2002). Although influential in areas such as reasoning, judgment, and decision-making, these models have sparked a debate about the cognitive processes that underlie intuitive and deliberate thinking and the extent to which they differ from each other (e.g., De Neys, 2021; Gawronski et al., 2014; Evans & Stanovich, 2013).

**Fig. 1:**
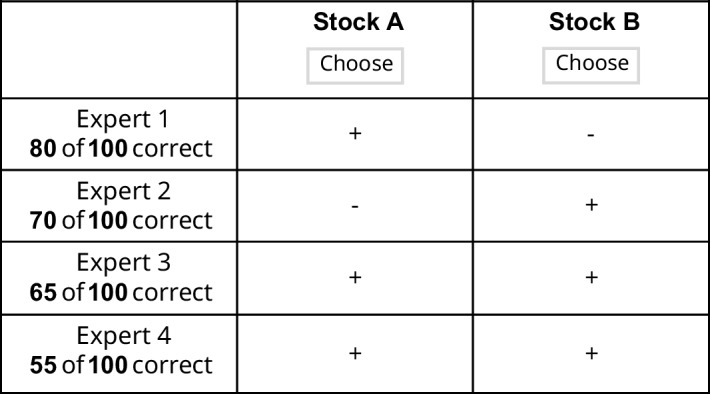
Example probabilistic inference task. A and B represent the two choice options. Experts (i.e., cues) provide dichotomous predictions (“+” = stock will perform well, “–” = stock will perform poorly). Cues are ordered by decreasing validity, which reflects how often each expert’s predictions were correct in the past (e.g., Expert 1 was correct in 80 out of 100 cases)

Defining intuition and deliberation solely by surface features such as speed or effort has been criticized as inadequate, as these features are not consistently observed, do not reliably co-occur, and reveal little about the underlying processes (Gawronski et al., 2014; De Neys, 2021; Melnikoff & Bargh, 2018). However, identifying the cognitive processes behind feature differences is complicated by the fact that they cannot be directly observed from the outside, and intuition is often defined by involving unconscious processes (Glöckner & Witteman, 2010), which limits access through verbalization or self-reporting.

A promising approach to translating dual-process theories into empirically testable hypotheses is the use of formalized cognitive models (Rumana, 2021; Glöckner, 2009). Such models specify precise, quantitative predictions for outcome measures such as judgments and decisions as well as process measures such as reaction times that can be compared across models and tested against empirically observable data (Marewski & Olsson, 2009; Guest & Martin, 2021). In the present study, we apply this modeling approach to assess whether the behavioral patterns observed under intuitive and deliberate conditions can be accounted for by adaptations within a common formalized mechanism, an integrated processes perspective (details below), or whether they are better captured by distinct models implying different underlying processes.

Task formats for which assumptions about intuitive and deliberate processing have been formalized within cognitive process models are therefore particularly well suited to test these competing perspectives. These include probabilistic inference tasks as shown in Fig. 1. In these tasks, participants judge which of two options is better on a not directly observable (distal) criterion based on probabilistic cue information (cue values) with a certain predictive validity (cue validity), such as expert predictions about stock performance (e.g., Glöckner et al., 2014, 2024; Newell & Shanks, 2003; Bröder, 2003). The open matrix format minimizes demands for information acquisition, allowing observation of behavior driven primarily by information integration processes.

## Cognitive process models of probabilistic inferences

**Fig. 2:**
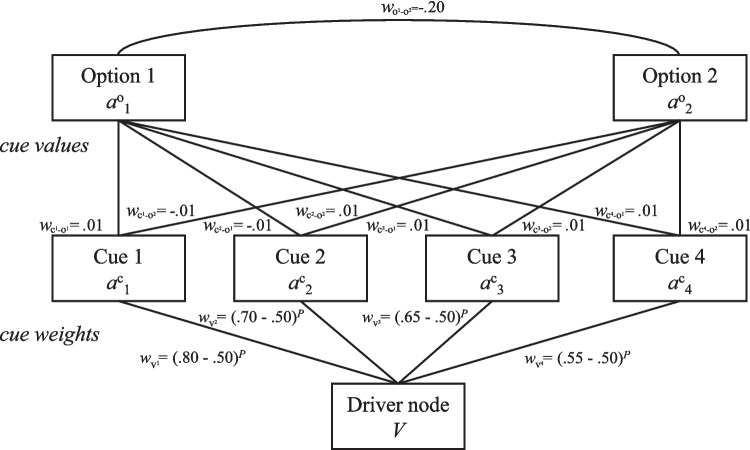
Network representation of the decision task from Fig. 1 in a PCS model. Connections between cue and option nodes, $w_{c-o}$, are excitatory or inhibitory depending on cue values, and cue nodes receive input weighted by subjective cue validities from a driver node that maintains network activation. The connections $w_{v_i}$ represent cue validities corrected for chance level (.50 in the case of binary choices between two options) and adjusted by parameter *P*. Through iterative, parallel updating of activation of nodes, $a^c$ and $a^o$, the network settles into a stable state of maximal coherence in which one option and its supporting evidence is favored. For a comprehensive description of the model, we refer to Glöckner and Betsch (2008a) and the Appendix B

As a candidate for a common underlying mechanism, we focus on the Parallel Constraint Satisfaction (PCS) network model of decision-making (Glöckner & Betsch, 2008a; Glöckner et al., 2014). It formalizes decisions as a (partly) automatic process of spreading activation among nodes representing cues and choice options (for a description, see Fig. 2 and the Appendix B). The class of PCS models has been proposed as a general account of various classes of cognition (McClelland et al., 2014, 2010). While originally developed to implement coherence-based mechanisms of perception (McClelland & Rumelhart, 1981; Rumelhart & McClelland, 1982; but see also Rumelhart et al., 1986), they have also been very successful in accounting for other processes such as social cognition (Kunda & Thagard, 1996; Read & Miller, 2014) and decision-making (Glöckner & Betsch, 2008a; Holyoak & Simon, 1999). Empirically, PCS models have outperformed competing accounts in explaining intuitive decision-making behavior (e.g., Heck and Erdfelder, 2017; Glöckner et al., 2024), including adaptations to environmental structure (Glöckner et al., 2014) and information search patterns (Jekel et al., 2018). On a theoretical level, the model assumes that decisions rely on partially automatic and partially unconscious integration of information to overcome limits of conscious capacity (Glöckner & Betsch, 2008a, 2012, 2008b), consistent with evidence that people integrate cues in a weighted, compensatory manner rapidly and effortlessly (Glöckner et al., 2024; Hochman, 2024; Brusovansky et al., 2018; Bröder, 2003). This perspective challenges a strict dichotomy that links intuition exclusively to non-compensatory and deliberation to compensatory information integration (Hochman, 2024; Ayal et al., 2011; Glöckner & Hochman, 2011).

In contrast, assuming a conscious, step-wise, rule-based information integration, according to the adaptive strategy selection view, limited cognitive resources encourage reliance on heuristics or rules of thumb that simplify the decision by ignoring information (Beach & Mitchell, 1978; Payne et al., 1988). Compensatory strategies in which positive and negative attributes are weighed so that strengths can offset weaknesses would therefore only be used for a few important slow decisions. In the present study, we include three formalized heuristics that differ in features relevant for investigating differences between intuitive and deliberative processing, namely in their complexity and degree of compensatory versus non-compensatory cue use (see also Glöckner et al., 2014; Glöckner et al., 2024; Pachur, 2022; Bröder, 2003; Glöckner and Hodges, 2011): the take-the-best heuristic (TTB), which bases decisions on the most valid discriminating cue (Gigerenzer & Goldstein, 1996); the equal weights strategy (EQW), which sums positive cues without weighting (Payne et al., 1988); and the weighted additive rule, which integrates cues by weighting them by change-corrected validity (WADD$_{\text {c}}$) (Payne et al., 1988; Jekel & Glöckner, 2018) (see Table 1 for a description of strategy predictions).

**Table 1:** Strategy predictions

Strategy		Prediction		
Abbreviation	Name	Choice	Confidence	Time
TTB	Take-the-best or lexiographic rule	Option favored by the most valid differentiating cue	Validity of the most valid differentiating cue	Number of elementary information processes required
WADD$_{\text {c}}$	Weighted additive corrected	Option with the highest chance-corrected weighted sum of cue values	Differences between chance-corrected weighted sums of cues	Number of elementary information processes required
EQW	Equal weight	Option with the highest unweighted sum of cue values	Difference between unweighted sums of cues	Number of elementary information processes required
PCS$_{\text {fix}}$	Parallel Constraint Satisfaction network model of decision-making with fixed parameter *P* = 1.9 and deterministic choices	Option with the highest final node activation of the option node	Absolute difference in the final activation between the option nodes	Number of iterations until convergence
PCS$_{\text {fitted}}$	Parallel Constraint Satisfaction network model of decision-making with per person fitted parameters *P* and $\lambda$	Option with the highest final activation of the option node, with determinacy governed by parameter $\lambda$	Absolute difference in the final activation between the option nodes	Number of iterations until convergence

### Comparability of intuitive and deliberate processes

Previous findings on probabilistic inferences do not allow conclusions to be drawn about the comparability of intuitive and deliberate decisions, as participants were usually instructed to respond quickly (e.g., Ayal & Hochman, 2009; Glöckner et al., 2024, 2014) or faced strict time limits (e.g., Brusovansky et al., 2018; Glöckner & Betsch, 2008b). Using an instruction to respond quickly facilitates interpretable response times (Fazio, 1990) but has also been proposed as a method to induce an intuitive processing mode (for reviews, see Horstmann et al., 2010; Isler & Yilmaz, 2022). According to the PCS model, the process of coherence maximization is always activated first but runs continuously and independently of the decision mode, while deliberate activity primarily monitors and adjusts the informational basis (i.e., the network structure) and supports the achievement of an acceptable level of coherence without changing the actual process directly (Glöckner & Betsch, 2008a). This represents an integrated processes view, according to which intuitive and deliberate decisions do not rely on entirely distinct processes but changes in the parameters of a single cognitive process (e.g., Glöckner & Betsch, 2008a; De Neys, 2023). In contrast, theories with a distinct processes view predict systematic shifts in strategy use under the instruction to deliberate, with more people relying on stepwise, rule-based processing (e.g., Sloman 1996; 2002; Petty & Cacioppo 1986; see Evans 2008, for a review). Initial support for the integrated processes view comes from eye-tracking evidence that shows that deliberation instructions, unlike instructions to apply rule-based calculations of weighted sums in line with a WADD strategy, do not qualitatively alter fixation duration patterns but primarily increase double-checking (Horstmann et al., 2009).

### Overview and research aim

In the present study, we employ a cognitive modeling approach to critically test competing predictions of the integrated and distinct processes view for intuitive and deliberate processing. Specifically, we examine (a) whether the distribution of strategies assigned to participants based on their choices, decision times, and confidence ratings differs between more intuitive versus more deliberate decision modes, and (b) whether existing models vary in their ability to capture the underlying decision processes across these modes. According to PCS representing the integrated processes view, the predictive accuracy of the PCS model should remain stable or even improve under deliberation, since deliberation efforts might be driven by the same factor (i.e., coherence) that has been shown to drive response time and confidence in intuitive decision-making (Glcökner & Betsch, 2012; see also Lee & Holyoak, 2021). Moreover, consistent with default-interventionist models (Evans, 2008), with deliberation more choices should align with a rational solution. This is due to the fact that the open matrix format used in the current study allows generating a mental (network) representation of the task that accurately represents the task structure, and deliberation can help to rule out remaining misinterpretations or mistakes (Glöckner & Betsch, 2008a). The rational solution in this case is a naïve Bayesian solution based on posterior probabilities assuming cue independence and equal priors (Lee & Cummins, 2004). By contrast, if intuitive and deliberate decisions rely on distinct processes, deliberation should promote shifts toward serial, rule-based, conscious heuristics, thereby reducing the fit of models grounded in parallel, (partially) automatic processing such as the PCS (Horstmann et al., 2009).

To test these predictions, we compare PCS and serial heuristics in accounting for decision behavior by deriving predictions not only for choices but also decision times, and confidence ratings (see Table 1). Unlike serial heuristics, PCS predicts decision times based on the time required to construct a coherent representation of information rather than the amount of integrated cue information, with additional information sometimes even speeding up decisions if it increases initial information coherence (Glöckner & Betsch, 2008a; Glöckner et al., 2014). Confidence is predicted from differences in option activations once a coherent solution is reached via bidirectional, parallel activation updating, whereas serial heuristics derive confidence from differences in support based on serial, unidirectional cue integration. Testing these predictions in a multi-measure approach allows differentiation between models and conditions that do not elicit unique choice responses, such as deliberate vs. intuitive processes (De Neys, 2023) or process models for probabilistic inferences (Glöckner, 2009; Jekel et al., 2010; Hilbig et al., 2010). It is sufficient if models differ in their predictions for process or other outcome variables. These variables impose additional constraints for more critical testing and efficient model refinement (Johnson et al., 2008; Jarecki et al., 2020).

## Method

The experiment was preregistered at the Open Science Framework (https://doi.org/10.17605/OSF.IO/D8BKZ). Hypotheses, data, experiment materials, and analysis code are available at the OSF project site (doi.org/10.17605/OSF.IO/7Q8MB).

### Design and manipulation

We used a 2 (DECISION MODE: Intuition vs. Deliberation) x 60 (TASK VERSION) design with TASK VERSION as a within-subjects factor. Decision mode was manipulated between participants. For this, we randomly assigned participants to two conditions (intuitive vs. deliberate) and varied instructions between these groups. In both conditions, participants were instructed to repeatedly choose the better of two options and to make good decisions (e.g., Glöckner et al., 2014). In the intuitive decision condition, participants were additionally asked to decide intuitively, spontaneously, and as quickly as possible. In contrast, in the deliberate condition, participants were asked to make conscious and reflected decisions and to take their time (original instructions in Appendix A). While a variety of experimental manipulations have been used to elicit intuitive versus deliberative decision-making modes (for overviews, see Horstmann et al., 2010; Isler & Yilmaz, 2022), the present approach is well established (e.g., Raoelison et al., 2021; Beauvais et al., 2025). It aims to accentuate process characteristics commonly linked to intuition and deliberation (Evans, 2008). It furthermore avoids the instruction of specific cognitive strategies, such as calculating weighted sums, or interference with parallel, partially unconscious processes through procedures like the loud verbalization of the decision-making process. In addition, the manipulation avoids confounds associated with alternative methods, such as emotion induction. Finally, no strict response deadlines are specified, preserving decision time as a dependent variable and allowing behavioral measures (choices, decision times, and confidence) to test model-based assumptions about cognitive processes as they occur without the imposition of specific strategies. To prevent regression to the mean (participants settling into less distinct modes over the course of the experiment), participants were reminded of these instructions in two breaks after every 20 trials.

### Participants

An a priori power analysis using a bootstrapping resampling method as well as G*Power (Faul et al., 2009) revealed a required total sample size of 128 participants to reach a power of at least $1-\beta =.80$ for each main hypothesis with $\alpha =.05$. A detailed description of the power analysis can be found in the Appendix D. Anticipating online experiment challenges such as participant exclusions, we collected 10% more than the required sample size and aimed for a total sample size of $N = 140$ participants. Correspondingly, we recruited a sample of *N* = 140 participants, of which *N* = 128 (81 female, 43 male, four other) with a mean age of 28.61 (*SD* = 10.20) fulfilled the criteria for inclusion. Participants were recruited from the Decision Lab Cologne participant pool and university mailing lists. The data collection was stopped after *N* = 140 complete data sets had been obtained. We only included complete data sets that allowed us to test our hypotheses. Additionally, persons who cancelled the task and then restarted it ($n = 6$) were excluded. As preregistered, we excluded participants who solved less than 55% of the tasks in accordance with the naive Bayesian solution ($n = 3$), assuming they did not conduct the study seriously, as well as people who indicated that they had not answered seriously ($n = 3$)^1^. These exclusions apply to all subsequent analyses. For the analysis of reaction times, we excluded trials with response times longer than 60 s, as well as data points exceeding three standard deviations from the grand mean across all participants, as preregistered (61 trials in total). The randomized assignment of participants to the two conditions and the participant exclusions resulted in 67 participants in the Intuition group and 61 participants in the Deliberation group.

The experiment lasted approximately 20 min. Participants from the participant pool were financially rewarded with a basic payment and a performance-dependent bonus. The other participants could receive course credit and take part in a lottery for a performance-dependent financial reward. All participants provided informed consent. Ethics approval was obtained from the German Association for Experimental Economic Research (Number FaGdgmFw, https://gfew.de/ethik/FaGdgmFw).

### Materials and procedure

The experiment was computer-based and conducted online. The participants were informed that they were taking part in a decision experiment. To ensure statistical power and data reliability, the experiment comprised 60 multi-cue probabilistic inference tasks. For this, 60 different combinations of cue patterns and cue validities were randomly generated (excluding dominated choices and duplicates). This avoidance of task repetition ensured that memory effects did not bias behavior toward fast, associative processing (e.g., activating the option previously selected in the same task), but left room for other strategies to operate. Adapted from previous research, the basic paradigm was a hypothetical stock market game (Bröder, 2003; Glöckner et al., 2014; Newell & Shanks, 2003). In this task, participants decided between two stocks (options) based on evaluations from four independent experts (cues) for which cue validities (predictive accuracies) were explicitly provided in an information matrix (see Fig. 1 and Table 2). The open matrix format minimizes the influence of information acquisition, such as reading additional information, and thus allows focusing on processes of information integration. Each matrix contained eight pieces of information (cue values) in the form of symbols indicating whether a good ("+") or bad ("-") performance is predicted for the respective stock in the coming month. Cue validity, presented as the proportion of 100 previous predictions of that expert that have come true, varied between the four experts and was presented below each cue label. Cues were sorted in descending order, with the most valid cue in the top row. To prevent influences of prior knowledge activated from memory (Dorrough et al., 2017), the options were labeled with “Stock A” and “Stock B” and the cues with “Expert 1” to “Expert 4”. Each probabilistic inference task was followed by an assessment of decision confidence, which was measured on a scale from very uncertain (50; i.e., random) to very certain (100; i.e., certain) using a horizontal slider. The 60 task versions were presented in randomized order and separated by two short breaks of self-paced duration after every 20 trials. All tasks were self-paced.

**Table 2:** Three examples out of 60 task versions used in the experiment

	Task 1			Task 2			Task 3		
Cue	$v_i$	A	B	$v_i$	A	B	$v_i$	A	B
1	.87	+	+	.94	+	+	.77	+	+
2	.87	+	–	.79	–	+	.74	+	–
3	.76	–	+	.73	+	–	.68	–	+
4	.60	–	+	.63	+	–	.63	+	+

*Note.* A and B represent the two choice options, with A defined as the option that has the higher posterior probability according to the naïve Bayesian solution, and $v_i$ denoting the validity of the respective cue. While the order in which the options were presented was randomized across trials, the labels “A” and “B” were always fixed to the left and right positions on the screen. This procedure ensured that the labels themselves did not provide information about which option was normatively superior

After the decision task, participants answered demographic questions and a questionnaire containing open and closed questions on the use of decision-making strategies in the previous task (see Appendix C). The study ended with a prompt asking participants to describe their strategy by moving several sliders to the right or left, with the ends labeled with opposite features typically associated with an intuitive or deliberate mode, derived from Evans (2008).

### Strategy predictions and classification

Choices, decision time, and confidence were predicted for all 60 probabilistic inference tasks given all considered strategies (PCS$_{\text {fix}}$, PCS$_{\text {fitted}}$, WADD$_{\text {c}}$, EQW, TTB). The approaches to derive the strategies’ predictions are explained in Table 1 and a further description of the strategies is provided in Appendices B and F.

In a fitted model version, PCS$_{\text {fitted}}$ contains up to two free person parameters. The sensitivity parameter *P* directly determines whether decisions are more compensatory ($P < 1$) or more non-compensatory with a higher sensitivity to differences in cue validities ($P > 1$) relative to a model that linearly weights cue validities ($P = 1$). A second free parameter, the determinism parameter $\lambda$, is relevant if a probabilistic version of the model is applied. Here, the predicted probability of choosing the preferred option for a given choice pattern follows a logistic choice function, with $\lambda$ determining the steepness of the function. For fitting these parameters, a grid-search fitting procedure was used to estimate the sensitivity parameter *P* in [0, 5]^2^ and the determinism parameter $\lambda$ in [0, 5] in steps of 0.1 to find the combination of parameter values that maximized the multiple-measure maximum log-likelihood of the observed data for each participant (Glöckner et al., 2014). Strategy predictions for choices, decision time, and decision confidence were then derived for the 60 different cue patterns according to the procedures described in Table 1 and transformed into contrast weights that add up to zero and have a range of one.

To determine the strategy that maximizes the likelihood of a participant’s data, we conducted a model classification with the multiple-measure maximum likelihood estimation method (MM-ML, Glöckner, 2009; Jekel et al., 2010). We calculated the maximum log-likelihood of observing the participants’ overall data vector, which includes their choices, decision times, and confidence ratings, given each potential strategy. Participants were then assigned to the strategy that yielded the smallest Bayesian information criterion (BIC) score, which penalizes strategies with a higher number of free model parameters (for a detailed description, see Glöckner et al., 2014). The inclusion of decision times and confidence judgments in the simultaneous MM-ML estimation allows differentiating between models that generate similar choice patterns but differ in their process predictions (Glöckner, 2009).

Statistical analyses were performed using R 4.3.2 (R Core Team, 2024), the lme4 (Bates et al., 2014), the lmerTest (Kuznetsova et al., 2017), and the glmmTMB (Brooks et al., 2017; McGillycuddy et al., 2025) packages. The significance level alpha was set at $\alpha =.05$. For all exploratory analyses, $\alpha$ was Bonferroni corrected with the number of exploratory tests (28) to $\alpha =.0018$. As in previous studies, decision times were ln-transformed and order effects were partialed out to reduce skewness and the influence of outliers (Glöckner et al., 2014).

**Fig. 3:**
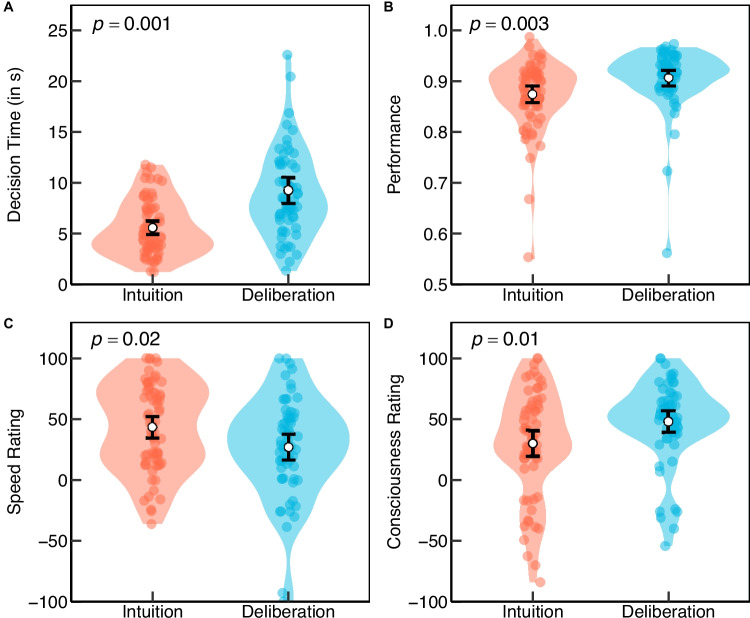
Behavioral and self-report differences between intuitive and deliberate decision modes. Decision time (**A**), performance (i.e., choices in line with the rational solution) (**B**), and self-report ratings concerning speed (**C**) and consciousness (**D**) of the applied decision strategy were averaged across trials for each participant. *Error bars* represent 95% confidence intervals of the mean

## Results

The manipulation of decision mode was effective, as indicated by both behavioral and subjective measures (Fig. 3). Participants in the deliberate condition exhibited significantly longer decision times ($M = 9241$ ms, $SD = 4917$ ms) than those in the intuitive condition ($M = 5583$ ms, $SD = 2694$ ms), $t(123.52) = 4.79$, $p <.001$, $d = 0.85$^3^. They also rated their strategy as more conscious ($M = 47.85$, $SD = 33.83$ vs. $M = 30.07$, $SD = 43.22$), $t(123.30) = 2.60$, $p =.010$, $d = 0.46$, and less fast ($M = 27.16$, $SD = 40.94$ vs. $M = 43.31$, $SD = 35.86$), $t(119.90) = 2.36$, $p =.020$, $d = 0.42$. Participants demonstrated the ability to solve the task with an average rate of 88.92 % correct decisions in accordance with the naïve Bayesian solution. As hypothesized, performance was significantly enhanced in the deliberate decision condition ($M = 0.91$, $SD = 0.06$) compared to the intuitive decision mode ($M = 0.87$, $SD = 0.07$), $t(125.99) = 2.84$, $p =.003$, $d = 0.50$.

Contrary to the hypothesized shift towards more serial strategies under the distinct processes view, choice adherence to PCS$_{\text {fitted}}$, defined as the proportion of choices aligning with strategy predictions averaged across participants, was significantly higher in the deliberate condition ($M = 0.95$, $SD = 0.04$) compared to the intuitive condition ($M = 0.91$, $SD = 0.13$), as determined by a non-parametric Mann–Whitney *U* test ($W = 1569$, $p =.022$).

**Table 3:** Choice adherence rates

		PCS Model Versions		Heuristics		
Condition	*N*	PCS$_{\text {fitted}}$	PCS$_{\text {fix}}$	WADD$_{\text {c}}$	EQW	TTB
Intuition	67	.91 (.004)	.87 (.005)	.88 (.005)	.82 (.008)	.85 (.006)
Deliberation	61	.95 (.004)	.90 (.005)	.91 (.005)	.80 (.008)	.89 (.005)
Overall	128	.93 (.003)	.89 (.004)	.89 (.004)	.81 (.006)	.87 (.004)

*Note.* *SE*s are in parentheses. For WADD$_{\text {c}}$ and EQW, the cue patterns for which random choice was predicted were excluded

**Fig. 4:**
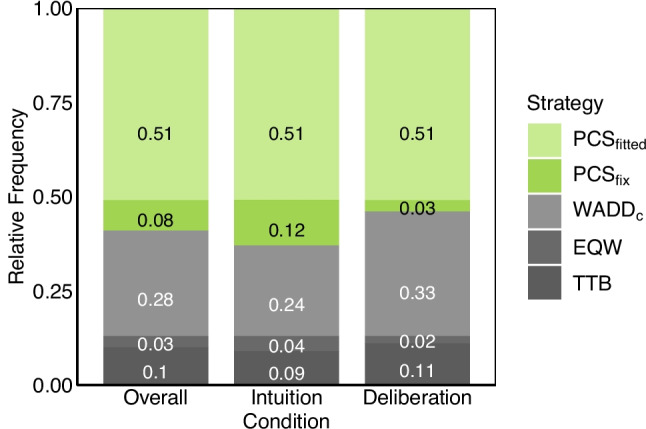
Strategy classification using a multiple measure strategy classification. All individuals could be classified as for no individual the best strategy showed a significant choice misfit compared to a saturated model (Glöckner et al., 2014). Percentages that do not add up to one are due to rounding

A joint analysis of choices, confidence ratings, and decision times using an MM-ML classification procedure (Glöckner, 2009) allowed participants to be assigned to a decision strategy. This was possible even though averaged choice adherence rates were above 80% for all strategies (Table 3). For most participants (59%), decision behavior was best explained by one of the two PCS implementations. An additional 28% were classified as using a compensatory WADD$_{\text {c}}$ strategy, whereas only 13% were assigned to simpler heuristics such as EQW or TTB (see Fig. 4). Importantly, there was no effect of decision mode on the distribution of strategy usage ($\chi ^2(4) = 4.99$, $p =.288$, Cramérs’ V $=.20$), suggesting comparable behavioral patterns across intuitive and deliberate conditions consistent with an integrated processes view.

As a robustness check and to reduce the risk of overfitting, we performed a six-fold cross-validation to investigate whether the strategy distribution changes if the PCS$_{\text {fitted}}$ model predicts new, unseen behavior (Hastie et al., 2009). Details on the procedure and detailed results can be found in Appendix E. The cross-validation procedure led to the same conclusions. There was a predominance of PCS classifications in both the intuitive and deliberate decision mode conditions (74% and 66%, respectively) and no indication of a strategy switch between them ($\chi ^2(4) = 1.86$, $p =.761$, Cramérs’ V $=.12$). The number of PCS classifications even increased compared to the previous classifications based on BIC values for both decision modes. This was mainly due to switches from WADD$_{\text {c}}$ to PCS$_{\text {fix}}$ and from PCS$_{\text {fitted}}$ to PCS$_{\text {fix}}$ classifications.

**Fig. 5:**
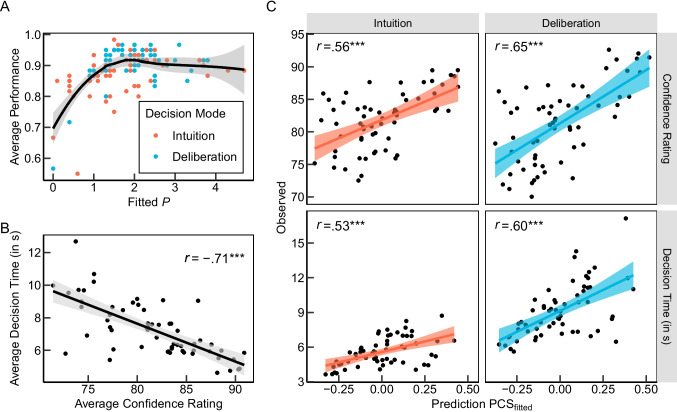
Associations between outcome and process measures and PCS$_{\text {fitted}}$ model predictions across decision modes. **A** Average performance (proportion correct) as a function of the fitted PCS model parameter *P*. *Points* represent participant-level data, with a LOESS smoother (*black line*) and 95% confidence band. **B** Association between average confidence ratings and decision times across the 60 tasks. **C** Observed response times, confidence ratings, and PCS$_{\text {fitted}}$ predictions for the 60 tasks. Coefficients *r* indicate Pearson correlations. For B and C, response times longer than 60 s and values exceeding three standard deviations from the mean were excluded. ***$p<$ .001

We found high positive correlations between PCS$_{\text {fitted}}$ predictions and decision times averaged across participants for both intuitive and deliberate decisions, as visualized in Fig. 5C. Consistent with PCS predictions, people decided faster and with greater certainty when a task had a clearer superior option that made it easier to form a coherent preference (see Fig. 5B). Individual-level correlations between strategy predictions and observed decision times, averaged using Fisher *z*-transformation, were highest for PCS$_{\textit{fitted}}$ predictions in both conditions (Table 4). Contrary to predictions of heuristics like EQW and WADD$_{\text {c}}$, decision times varied systematically across tasks, with longer times required for a priori less coherent information patterns with a less clearly superior option. A linear mixed model (LMM) analysis with random intercepts at the participant level confirmed a significant positive association between PCS$_{\text {fitted}}$ predictions and observed decision times ($b = 0.48$, $SE = 0.04$, $t(7489) = 13.56$, $p <.001$). A main effect of dummy-coded decision mode (0 = Intuition, 1 = Deliberation) again indicated longer decision times in the deliberate condition ($b = 0.46$, $SE = 0.10$, $t(126) = 4.79$, $p <.001$). Crucially, a significant interaction between decision mode and PCS$_{\text {fitted}}$ predictions indicated that the association of PCS$_{\text {fitted}}$ predictions with observed decision times was more pronounced in the deliberation condition than in the intuition condition ($b = 0.17$, $SE = 0.05$, $t(7489) = 3.33$, $p <.001$), contrary to predictions of the distinct processes account.^4^

**Table 4:** Individual-level correlations between predicted and observed decision time and confidence

		PCS Model Versions		Heuristics		
Condition	*N*	PCS$_{\text {fitted}}$	PCS$_{\text {fix}}$	WADD$_{\text {c}}$	EQW	TTB
*Decision Time*						
Intuition	67	.23	.23	(0)	(0)	-.04
Deliberation	61	.29	.27	(0)	(0)	-.04
Overall	128	.25	.25	(0)	(0)	-.04
*Confidence*						
Intuition	67	.29	.25	.30	.05	.15
Deliberation	61	.38	.36	.41	.05	.21
Overall	128	.33	.31	.35	.05	.18

*Note.* Correlations were calculated per individual and then averaged using Fisher *z*-transformation. Correlations averaged across cue patterns for PCS$_{\text {fitted}}$ are reported in Fig. 5

A similar pattern was found for confidence ratings. As shown in the lower part of Table 4, confidence ratings were most strongly associated with PCS predictions and predictions of WADD$_{\text {c}}$. In an LMM with random intercepts at the participant level, PCS$_{\text {fitted}}$ predicted confidence ratings very well ($b = 10.69$, $SE = 0.61$, $t(7550) = 17.62$, $p <.001$). There was no significant main effect of decision mode on confidence ($b = -0.52$, $SE = 1.54$, $t(126) = 0.34$, $p =.738$). Crucially, a significant interaction between decision mode condition and PCS$_{\text {fitted}}$ predictions ($b = 4.99$, $SE = 0.87$, $t(7550) = 5.71$, $p <.001$) again revealed that PCS$_{\text {fitted}}$ predicted confidence ratings in the deliberate condition better than in the intuitive condition, contrary to predictions of the distinct processes view^5^.

As a further robustness check, we analyzed whether the results remained the same when the parameter range is restricted to a smaller range of *P* = [1, 2], as originally pre-registered. Although such a restriction leads to considerable problems with corner solutions (e.g., 58% of persons have parameter values of 1 or 2), the conclusions concerning all hypotheses remained unchanged. Detailed results are reported in the OSF project.

To investigate potential modifications within the PCS process by the decision mode manipulation, we compared average values of PCS parameter *P* between the intuitive and deliberate conditions in several preregistered exploratory analyses. *P* captures intra- and inter-individual differences in the scaling of and the sensitivity to differences in cue validities (for details, see Appendix B). Descriptively, the average fitted *P* parameter was higher for deliberate decisions ($M = 1.97$, $SD = 0.81$) compared to intuitive decisions ($M = 1.70$, $SD = 0.95$) (see Fig. 5A), although not significant at the Bonferroni-corrected $\alpha$ level ($U = 1612$ $p =.039$) in a non-parametric Mann–Whitney *U* test.

## Discussion

This work used a multi-measure cognitive modeling approach to compare competing predictions of whether the same cognitive models can account for behavior under both intuitive and deliberate decision modes, or whether distinct models better capture behavior in each condition. We found a higher choice quality, longer decision times, and a subjective experience of conscious decision-making under the instruction to deliberate. However, a PCS model of parallel, compensatory information integration accounted for choices, decision time, and metacognitive decision confidence in both decision modes. Further analyses that classified participants to their most likely decision strategy based on all three measures found no difference in strategy distribution between decision modes, with 50–60% being classified to a PCS strategy, fewer than 30% to a weighted additive strategy (WADD$_{\text {c}}$), and less than 15% to simpler heuristics (TTB, EQW). Both findings align with self-reported differences in speed and effort but not in process characteristics such as associative versus rule-based or sequential versus parallel thinking (see Table 8), highlighting that several attributes commonly associated with intuition and deliberation do not necessarily co-occur (Melnikoff & Bargh, 2018; but see also Evans & Stanovich 2013).

Arguing against demanding serial integration of weighted cues, we observed generally short latencies and faster responses when cue predictions aligned, despite a constant number of cues. This finding is incompatible with serial, conscious integration models such as WADD$_{\text {c}}$. Participants classified as WADD$_{\text {c}}$ users may therefore have partially relied on PCS processes that approximate the rational integration of all available information assumed by WADD$_{\text {c}}$ (Brusovansky et al., 2018; Horstmann et al., 2009; Glöckner & Betsch, 2008b). This conclusion aligns with the observation that participants tended to disagree with having deliberately used formula-based strategies in our questionnaire (Table 7) and with the substantial number of classification switches from WADD$_{\text {c}}$ to PCS in the cross-validation. It is also consistent with the dominance of short fixations even under the instruction to deliberate in a previous study, in contrast to a pattern of particularly long fixations observed under the instruction to consciously calculate weighted sums (Horstmann et al., 2009). Strategy classification results of the present study may therefore benefit from further validation studies by combining cognitive modeling approaches with more direct process-tracing methods (e.g., Seitz et al., 2025).

Beyond strategy classifications based on relative model fit, the close absolute correspondence between PCS predictions and observed behavior supports theoretical claims that a single unified parametric model can account for both intuitive and deliberate behavior and therefore the idea of integrative dual-process models (e.g., Keren & Schul, 2009; Kruglanski et al., 2006; Braem et al., 2023; Kruglanski & Gigerenzer, 2011; Osman, 2004). Importantly, testing predictions derived from formalized parametric models allows research to move beyond questions about the number of cognitive systems. Instead, it enables explicit tests of assumptions about the nature of intuitive and deliberative processes and their interaction, as advocated in contemporary dual-process research (De Neys, 2021, 2025; Glöckner & Witteman, 2010). In the present study, findings of the predicted effects of initial information coherence on decision time and confidence suggest that coherence-structuring processes that detect and resolve conflict among cognitions to form coherent representations (good "Gestalts”) may underlie both intuitive and deliberate processing (see also De Neys, 2014). In line with conflict-monitoring accounts of cognitive control (Botvinick et al., 2001), such monitoring and resolution of conflict and uncertainty is often attributed a central role in the initiation and function of deliberation in contemporary dual-process frameworks (e.g., De Neys, 2023; Pennycook et al., 2015; De Neys, 2025; Cho et al., 2023; Braem et al., 2023; Evans & Stanovich, 2013), but have remained insufficiently specified. The present work suggests that these processes can be formalized as coherence maximization within a connectionist network and operate independently of decision mode. However, when coherence falls below a subjective threshold, or people are forced by explicit instruction, deliberation can potentially induce changes in specific model parameters to further support conflict resolution (Glöckner & Betsch, 2008a). These may involve the construction of the network and thus information representation^6^, the dynamics of bidirectional activation updating and thus coherence maximization (cf. Lee & Holyoak, 2021), or more complex constructive processes that serve to double-check or improve initial interpretations (cf. Horstmann et al., 2009). Using the terminology of Stanovich’s tripartite model of the mind (Evans & Stanovich, 2013; Stanovich, 2009, 2011, 2009b), our manipulation has activated control processes of the reflective mind that might have involved processes of control and decoupling. The manipulation has not activated qualitatively different processes of the algorithmic mind (but also does not speak against their existence). Importantly, depending on the specific processes amplified by deliberation, increased effort does not necessarily result in more normative decisions (De Neys, 2025). Nevertheless, coherence processes and all processes that serve this goal are adaptive in themselves in enabling confident action in the face of extreme ambiguity and conflicting information (Simon & Read, 2023; Simon et al., 2015; Holyoak & Simon, 1999).

Other single parametric models, such as evidence accumulation models (EAMs), conceptualize differences between intuitive and deliberate processing as parameter adaptations in sequential evidence sampling (Braem et al., 2023; Li et al., 2024; Alós-Ferrer, 2018; Cho et al., 2023). The intuition and deliberation instructions used in this and previous studies (e.g., Raoelison et al., 2021; Beauvais et al., 2025) partially overlap with speed–accuracy trade-off manipulations that increase response thresholds (Heitz, 2014). Such threshold adjustments could account for higher performance and longer decision times under deliberation (Heitz, 2014; Braem et al., 2023). However, we found no differences in decision confidence and (non-)compensatory cue integration between conditions typically associated with threshold differences (e.g., Lee & Cummins, 2004; Söllner & Bröder, 2016; Hausmann & Läge, 2008; Lee et al., 2023). Additionally, effects of initial coherence on decision time and confidence observed here and in prior work (e.g., Glöckner & Betsch, 2012) and the finding that individuals typically change the evaluation of the evidence in the decision process instead of merely accumulating it (i.e., coherence effects also referred to as pre-decisional information distortions, Russo, 2015; Simon & Read, 2023; Glöckner et al., 2010) are more directly explained by PCS and require additional assumptions in sequential, unidirectional EAMs (e.g., Pleskac & Busemeyer, 2010; Wang et al., 2024). However, since EAMs can capture some aspects of the data, future research might investigate whether threshold adaptations account for differences between intuitive and deliberate instructions in other tasks or task formats.

Overall, our study shows that PCS-like mechanisms provide the best account of individuals’ behavior across both intuitive and deliberate conditions, despite differences in performance, response time, and subjective measures. This is consistent with recent advances in applying coherence accounts as a parsimonious approach to higher cognitive functions (McClelland et al., 2010) and as a common explanation of various biases (Simon & Read, 2023; Oeberst & Imhoff, 2023). Also, our results support the broader claim that connectionist architectures capture core principles of neural information processing (Simon & Read, 2023). Importantly, this convergence does not warrant the general conclusion that identical processes underlie intuitive and deliberate decisions. First, strategy classification is inherently limited to the set of models considered, leaving open the possibility that untested mechanisms could produce similar patterns (Glöckner & Bröder, 2011). Future work should therefore include additional candidate models, including EAMs that have also been shown to effectively explain choices and decision times in other decision tasks (Ratcliff et al., 2015; Milosavljevic et al., 2010; Ratcliff et al., 1999). Importantly, the use of multiple dependent measures largely reduces the risk that the behavioral pattern was produced by other models not included in the model comparison (Jarecki et al., 2020). Second, other deliberation manipulations or task formats that involve sequential or effortful information processing may engage qualitatively different processes (Horstmann et al., 2009; Söllner et al., 2013; Bröder & Schiffer, 2003; Bröder & Gaissmaier, 2007). Taking these considerations into account, we conclude that for this specific and widely used manipulation of decision mode and task format investigated in our study, PCS-like mechanisms best capture the observed behavioral patterns. Methodologically, our results highlight the value of comparing intuitive and deliberate behavior using cognitive process models and multiple dependent measures, including response time as a process measure and metacognitive confidence, to more stringently test assumptions of dual-process theories.

## Data Availability

The experiment was preregistered at the Open Science Framework (10.17605/OSF.IO/D8BKZ). The anonymized data, the experiment materials, the complete analysis code, and all results are available at the OSF project site (10.17605/OSF.IO/7Q8MB).

## Appendix A: Original instruction

**Table 5:** Decision mode instructions

Condition	Beginning	Breaks
Intuition	Important! Decide intuitively, spontaneously and as quickly as possible [Wichtig! Entscheiden Sie intuitiv, spontan und so schnell wie möglich]	Reminder: In each round, your task is to select the stock that you think is better. Decide intuitively, spontaneously and as quickly as possible. [Zur Erinnerung: In jeder Runde ist Ihre Aufgabe, die Aktie auszuwählen, die Sie für besser halten. Entscheiden Sie dabei intuitiv, spontan und so schnell wie möglich.]
Deliberation	Important! Make a conscious, reflective decision and take your time. [Wichtig! Entscheiden Sie bewusst, reflektiert und nehmen Sie sich Zeit.]	Reminder: In each round, your task is to select the stock that you think is better. Make a conscious, reflective decision and take your time. [Zur Erinnerung: In jeder Runde ist Ihre Aufgabe, die Aktie auszuwählen, die Sie für besser halten. Entscheiden Sie dabei bewusst, reflektiert und nehmen Sie sich Zeit.]

*Note.* Participants were reminded of the instructions in two breaks after every 20 trials. The complete instructions can be found on OSF

## Appendix B: PCS model specification

The PCS algorithm is modeled as a repeated simultaneous updating of the activation of the nodes within the network, using a sigmoid activation function proposed by McClelland and Rumelhart (1981):

1 $$\begin{aligned} \begin{aligned} a_i(t + 1)&= a_i(t)(1 - \text {decay}) \\&\quad + {\left\{ \begin{array}{ll} \text {input}_i(a_i(t) - \text {floor}), & \text {if input}_i < 0 \\ \text {input}_i(\text {ceiling} - a_i(t)), & \text {if input}_i \ge 0 \end{array}\right. } \\ \text {with} \\ \text {input}_i(t)&= \sum _{j = 1\rightarrow n} w_{ij} a_j(t) \end{aligned} \end{aligned}$$

**Table 6:** Specified and free model parameters

Parameter	Value/function	Comment
*Decay*	0.10	Constant decay factor determining the decay of node activation over time
$w_{o1-02}$	-0.20	Inhibitory connection between options
$w_{c-o}$	0.01/-0.01	Weight of the links between cues and options representing positive or negative predictions
$w_v$	$w_v = (v-0.50)^P$	Weight of the links between cues and driver node representing cue validities corrected for chance level and adjusted by parameter *P*
*Ceiling*/*floor*	1/-1	Minimum and maximum possible activation
*P*	Free	Sensitivity parameter. Represents subjective differences in the scaling of and sensitivity to cue validities. Can be fixed to *P* = 1.9 for the PCS$_{\text {fix}}$ version of the model
$\lambda$	Free	Determinism parameter in the choice function in the probabilistic $_{P}$PCS version of the model

In this function, $a_i(t)$ represents the activation of the node *i* at iteration *t*. With each iteration, this activation changes due to a decay factor and $input_i(t)$. The decay factor is set to a constant value of $decay =.1$ and determines the decay of node activation over time. $Input_i(t)$ is the activation that node *i* receives at iteration *t* and is calculated by summing the products of the activation of the nodes connected to node *i* and the weights $w_{ij}$ on this connection. The minimum and maximum possible activation are represented by *ceiling* and *floor*. This updating PCS process maximizes the information coherence under parallel consideration of all constraints (Glöckner et al., 2014). The overall coherence in the network can be quantified with the energy in the network, calculated by:

2 $$\begin{aligned} Energy(t) = -\sum _{i}\sum _{j}{w_{ij}a_ia_j} \end{aligned}$$

Stability is considered to be achieved when the changes in the overall energy are below a threshold value of $10^{-6}$ for more than ten iterations. Once stability is reached, the update process ends.

By specifying the precise structure of the network and several parameters (see Table 6), the model’s degrees of freedom were reduced to zero, one or up to two free parameters (Glöckner et al., 2014; Glöckner & Betsch, 2008a). Parameter *P* captures intra- and inter-individual differences in the scaling of and the sensitivity to differences in cue validities (Glöckner & Betsch, 2008a). In a Monte Carlo simulation with randomly generated tasks and sets of validities in a four-cue environment, a parameter value of $P = 1.9$ led to most choices in line with the Naïve Bayesian solution (Glöckner et al., 2014). Another comprehensive simulation found similar results, with a maximum alignment in predictions of 88.8% with *P* = 2 (Jekel et al., 2014). In the present study, fixing *P* at 1.9 results in the PCS$_{\text {fix}}$ version of the model, which has no free parameters and enables model predictions without empirical data. A second free parameter, the determinism parameter $\lambda$ in the choice function, is relevant if a probabilistic version of the model $_{P}$PCS is applied. In this model version, it is not assumed that the option with the higher final activation is always selected, but that the probability for choosing the preferred option for choice pattern *t* follows a logistic choice function:

3 $$\begin{aligned} p[x = \textit{preferred}|P,\lambda ,t] = \frac{e^{\lambda {a ^o}_{p(t)}}}{e^{\lambda {a^o}_{p(t)}} + e^{\lambda {a^o}_{np(t)}}} \end{aligned}$$

with ${a^o}_p$ and ${a^o}_{np}$ indicating the activations for the preferred and the non-preferred option (in the two-option case) and $\lambda$ indicating the steepness of the choice function (Glöckner et al., 2014).

## Appendix C: Self-descriptions of the applied strategies

In preregistered exploratory analyses, we examined whether participants’ descriptions of their decision strategies differed by instruction-induced decision mode. Open-ended responses can be found on OSF. The closed questions were taken from a previous study (Glöckner & Engel, 2013) and adapted to the context of this experiment. Agreement to the closed questions was rated on a scale from 1 (do not agree at all) to 5 (fully agree). We used eight non-parametric Mann–Whitney *U* tests to compare participants’ agreement with statements about decision strategies between the intuitive and deliberate conditions, due to violations of normality assumptions (Table 7). No significant differences were found at the Bonferroni-corrected alpha level.

**Table 7:** Results of decision strategy questionnaire

	Items	$M_{\text {Int}}$	$M_{\text {Del}}$	*U*	*p*
1	I used all available information in my decision.	3.58	4.11	1,524.00	.010
2	I applied mathematical formulas to calculate which option was better.	1.87	1.95	1,891.00	.425
3	I took into account the reliability of all experts.	3.66	4.23	1,508.50	.007
4	I first tried to detect the most reliable piece of information and then I decided solely on the basis of this information.	3.06	3.07	2,057.00	.949
5	I added up the number of predictions for and against an option and decided in favor of the option with the higher sum.	2.84	2.77	2,098.00	.792
6	I weighted the information for and against an option with its reliability, added them up, and decided for the option with the higher weighted sum.	3.09	3.15	1,993.00	.806
7	I checked the overall argumentation for and against both options for consistency.	2.81	3.20	1,691.00	.085
8	I tried to find the best possible interpretation of the provided information.	4.10	4.28	1,957.50	.656

*Note.* M$_{\text {Int}}$ and M$_{\text {Del}}$ are the mean agreement scores in the intuitive and deliberate group. Note the limited interpretability of the mean value, as the five-point Likert scale may be ordinal rather than interval-scaled. Significance tests result from Mann–Whitney *U* tests testing for differences between both groups. Significance levels were Bonferroni corrected to *p* = .0018

Next, we explored whether participants used different attributes typically associated with intuitive or deliberate decision-making to describe their strategies. Ten exploratory two-tailed two-sample *t* tests were conducted, one for each attribute pair, to examine differences in slider positions across conditions (Table 8). No significant differences were found between groups at the Bonferroni-corrected alpha level. This finding was robust to violations of normality, as confirmed by non-parametric Mann–Whitney *U* tests.

**Table 8:** Attribute slider ratings compared between conditions

Attribute pair		Results			
Deliberation	Intuition	$M_{\text {Int}}$	$M_{\text {Del}}$	*t*	*p*
Controlled	Automatic	-13.07	-34.89	2.98	.003
High effort	Low effort	24.00	5.18	2.66	.009
Analytic	Holistic	-0.85	-5.46	0.58	.560
Linked to language	Nonverbal	41.49	50.49	-1.11	.269
Rule-based	Associative	-17.28	-18.52	0.16	.873
Abstract	Contextualized	11.90	14.61	-0.31	.754
Logical	Pragmatic	-17.45	-22.62	0.68	.497
Sequential	Parallel	18.93	6.77	1.61	.109

*Note.* The ends of the slider were labeled with two opposing attributes that are typically associated with deliberation or intuition (left part). The attribute displayed on the right-hand side of the slider was randomized across participants, and the numerical slider values were not visible to the participants. $M_{\text {Intuition}}$ and $M_{\text {Deliberation}}$ represent the mean ratings in the intuitive or deliberate condition (right part). A negative value indicates that the mean rating was closer to the attribute associated with deliberation. Statistics result from *t* tests for two independent samples. Significance levels were Bonferroni corrected to $p = .0018$

To examine whether participants’ self-reported strategy attributes correlated with confidence, decision time, or performance, we conducted multiple linear regressions using ratings of ten strategy attribute pairs as predictors. A significant effect of the slow-fast rating emerged for decision time ($b = -0.01$, $SE = 0.00$, $t = 3.94$, $p <.001$), indicating that participants who rated their strategy as faster made quicker decisions. Similarly, the slow-fast rating significantly predicted confidence ($b = 0.09$, $SE = 0.02$, $t = 3.84$, $p <.001$), with faster strategies associated with higher confidence. No significant effects were observed for other attributes or for decision performance at the Bonferroni-corrected alpha level.

## Appendix D: Power analysis

Power analyses for multi-level analyses were conducted using simulations. For predicting decision time and confidence ratings using PCS predictions, we created 1000 bootstrap datasets by resampling *N* participants with replacement out of a comparable dataset (Glöckner et al., 2014, Experiment 1). For each dataset, a multilevel random intercept regression was conducted to predict confidence ratings using PCS$_{\text {fix}}$ model predictions, while controlling for cue patterns and environment. Power was calculated based on the proportion of significant *p* values ($p <.05$) in 1000 bootstraps, aiming for a power greater than $1-\beta =.80$. Due to the previously observed substantial effects and the repeated measurement design, this power analysis showed a high power ($1-\beta =.884$) even with very small sample sizes ($N = 10$). For comparing adherence rates (proportion of choices in line with PCS predictions) between the intuitive and deliberate groups, a G*Power analysis (Faul et al., 2009) for a two-tailed *t* test for two independent groups ($\alpha =.05$) indicated a required sample size of 128 participants (i.e., 64 per group), assuming a medium effect size of $d = 0.50$ as the smallest effect of interest. All G*Power analyses were a priori analyses conducted with $1-\beta =.80$ and $\alpha =.05$. Considering that a higher performance in the deliberate condition is also tested with a two-sample *t* test but one-tailed, the power of this analysis is higher. For comparing strategy use distribution using a $\chi$-squared test, the G*Power analysis indicated that $N = 122$ participants are needed, assuming a medium effect size of Cramer’s V $= 0.30$ $(df = 3)$. In conclusion, 128 participants is the highest required number of participants to test our hypotheses.

## Appendix E: Cross-validation

To assess the generalizability of the strategy classification results, we performed a six-fold cross-validation at the participant level. For each participant, the 60 experimental trials were randomly partitioned into six folds of equal size (ten trials each). In each iteration, one fold was held out as the validation set, while the remaining five folds (50 trials) served as the training set for estimating the free model parameters, namely the sensitivity parameter *P* and the determinism parameter $\lambda$. Parameter estimation was conducted on the training data only, and the resulting fitted values were then used to generate PCS predictions for the held-out validation set. This procedure was repeated until each fold had served once as the validation set, ensuring that every trial contributed to both model fitting and out-of-sample evaluation. Multi-measure log-likelihood values were computed for each fold and summed over the k folds for each participant and strategy to then classify participants to the most likely decision strategy based on this cross-validated log-likelihood.

**Table 9:** Cross-validation transitions in strategy classification

	Classification in six-fold cross-validation (robustness check)					
Classification using BIC correction (see main text)	PCS$_{\text {fitted}}$	PCS$_{\text {fix}}$	WADD$_{\text {c}}$	EQW	TTB	Total
PCS$_{\text {fitted}}$	35	20	6	0	4	65
PCS$_{\text {fix}}$	0	10	0	0	0	10
WADD$_{\text {c}}$	5	17	12	0	2	36
EQW	1	0	0	2	1	4
TTB	2	0	0	0	11	13
Total	43	47	18	2	18	128

*Note.* Rows represent strategy classifications obtained from fitting PCS$_{\text {fitted}}$ with the whole dataset. Columns represent classifications obtained via six-fold cross-validation. Diagonal cells indicate consistent classifications across methods. Row and column totals indicate overall counts

## Appendix F: Heuristics included in the analyses

This appendix provides a description of the decision heuristics included in the analyses following the formulations provided by Glöckner and Hodges (2011). The approaches used to derive each strategy’s predictions are summarized in Table 1.

According to a Take-The-Best (TTB) heuristic (a special case of LEX), individuals only retrieve information on the most valid cue and select the option that scores higher on this cue. The second cue is retrieved only if the first cue does not differentiate between options, and so on for the third cue, etc.

The Equal-Weight (EQW) heuristic assumes that people only add up positive and negative cue values of both options and choose the option with the higher sum.

The deliberate Weighted Additive (WADD) strategy assumes that individuals calculate the weighted sum of cue values and cue validities for each option and select the option with the higher weighted sum.

## Footnotes

1 Robustness analyses indicated that the overall pattern of results was unaffected by exclusion criteria. Including all participants regardless of seriousness checks or choice behavior yielded comparable results, with only minor shifts in the distribution of strategies. Of the six participants excluded, three were classified as WADD$_{\text {c}}$ and three as EQW users

2 Since an accumulation of fitted *P* parameter values was observed at the upper and lower limits of the parameter space, the parameter space was extended from [1, 2] to [0, 5] in deviation from the preregistration. The results with the original parameter space do not deviate substantially from the reported results and are available at the OSF project site.

3 This result, obtained from a Welch’s two-sample *t* test, remained significant after excluding the first ten trials of initial task familiarization. The same also holds when replacing the aggregated analysis with a linear mixed-effects model including random intercepts at the item level, as it is also the case for the differences in performance and adherence to PCS$_{\text {fitted}}$ predictions reported below

4 For LMM, the pattern of significance remained the same when the PCS fitting process was performed without the respective variable to be predicted (for details, see results on OSF).

5 These results were corroborated by a generalized linear mixed-effects model with a beta distribution, which accounted for the bounded nature of confidence ratings and violations of normality and homoscedasticity (Brooks et al., 2017; Geissinger et al., 2022)

6 With respect to network construction, exploratory analyses suggest that deliberation may shift *P* toward its optimal value of $P \approx 2$, which yielded maximal correspondence (88.8%) between the PCS and a normative naïve Bayes solution in prior simulation studies (Jekel et al., 2012, see Appendix B). Under this interpretation, the PCS approximates naïve Bayes through partially automatic, parallel processing rather than computationally demanding conscious calculations, and this approximation may be further strengthened by deliberation (Jekel et al., 2012; Glöckner et al., 2014).
